# Surgical Management of Perigraft Seroma by Graft Wrapping with Local Hemostatic Agents: A Case Report

**DOI:** 10.3400/avd.cr.22-00051

**Published:** 2022-09-25

**Authors:** Hiroshi Furukawa, Noriyasu Masuda, Kazuhiko Uwabe

**Affiliations:** 1 Department of Cardiovascular Surgery, Tokyo Women’s Medical University Adachi Medical Center, Tokyo, Japan

**Keywords:** perigraft seroma, polytetrafluoroethylene (PTFE), local hemostatic agent

## Abstract

A 76-year-old female developed progressive local groin bulging. She received regular hemodialysis using a left-thigh polytetrafluoroethylene arteriovenous graft in the loop configuration. Lower extremity enhanced computed tomography showed a large low-density area around the graft 18 months after its creation, and perigraft seroma (PS) was suspected. The patient underwent PS excision followed by graft wrapping with two local hemostatic agents, oxidized regenerated cellulose, and a fibrin sealant. Local PS recurrence was not detected four months after surgery. We herein describe a surgical case of refractory PS successfully treated by graft wrapping using two local hemostatic agents.

## Introduction

Perigraft seroma (PS) is local fluid collection around a vascular graft.^1)^ Its prevalence is reportedly 1.2%–2.4% after extra-anatomical bypass^2–4)^ and 1.7% after arteriovenous graft (AVG) placement for hemodialysis (HD).^5)^ Since PS is rare, there is no consensus on treatment strategies and management plans. PS may induce graft thrombosis, skin erosion, and secondary graft infection, ultimately resulting in graft failure.^5)^ We present a surgical case of PS that was successfully treated by graft wrapping using two types of local hemostatic agents, and discuss and suggest clinical strategies and management plans following prosthetic graft implantation.

## Case Report

**Figure 1:**
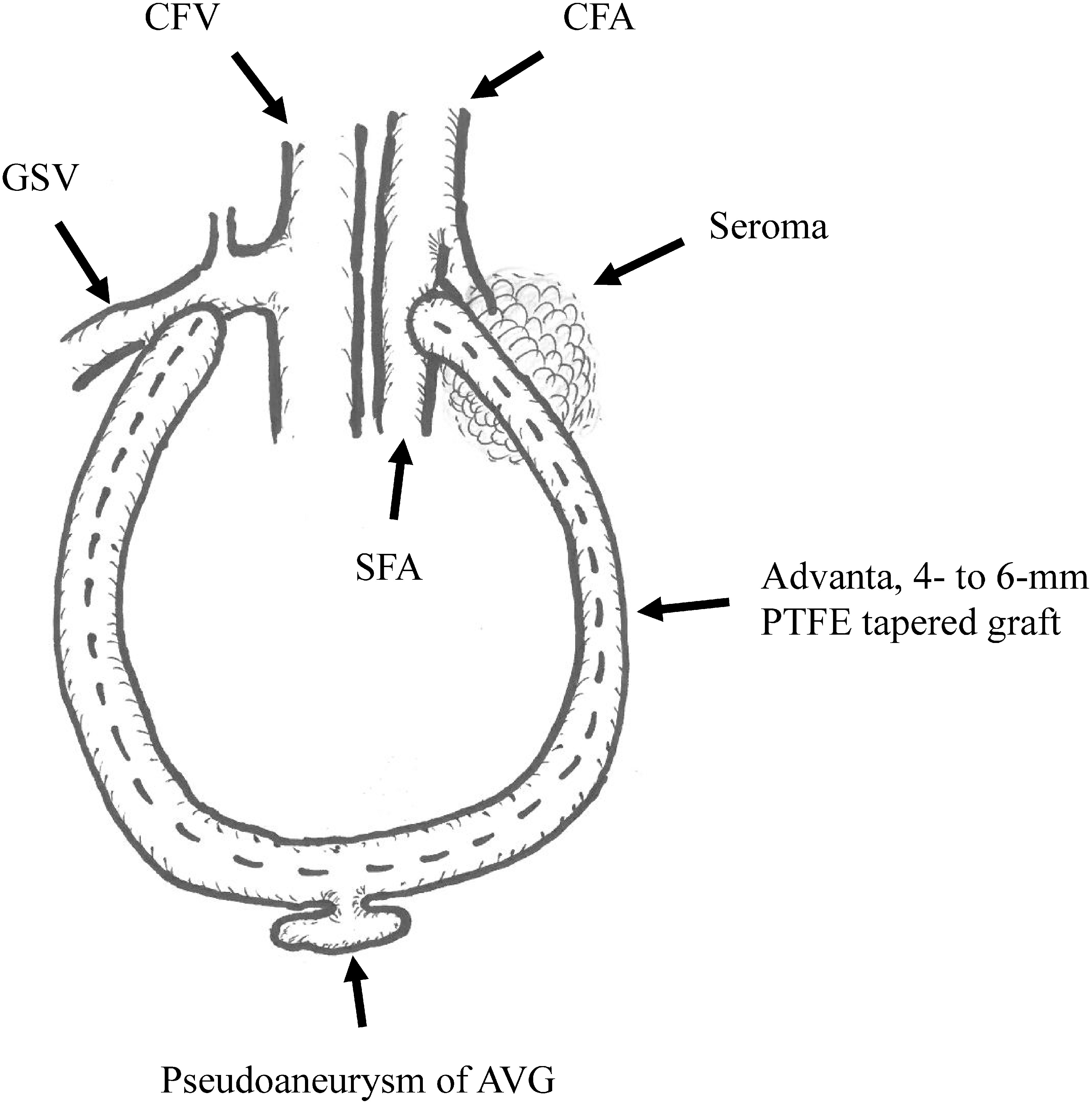
Fig. 1 Schema of thigh loop arteriovenous graft and locations of seroma and pseudoaneurysm of the graft. Thigh loop arteriovenous graft (AVG) was perfused from the proximal side of the superficial femoral artery to the proximal side of the great saphenous vein. Perigraft seroma was located just above the arterial side of anastomosis. A pseudoaneurysm of the graft was recognized at the tip of the AVG loop.

**Figure 2:**
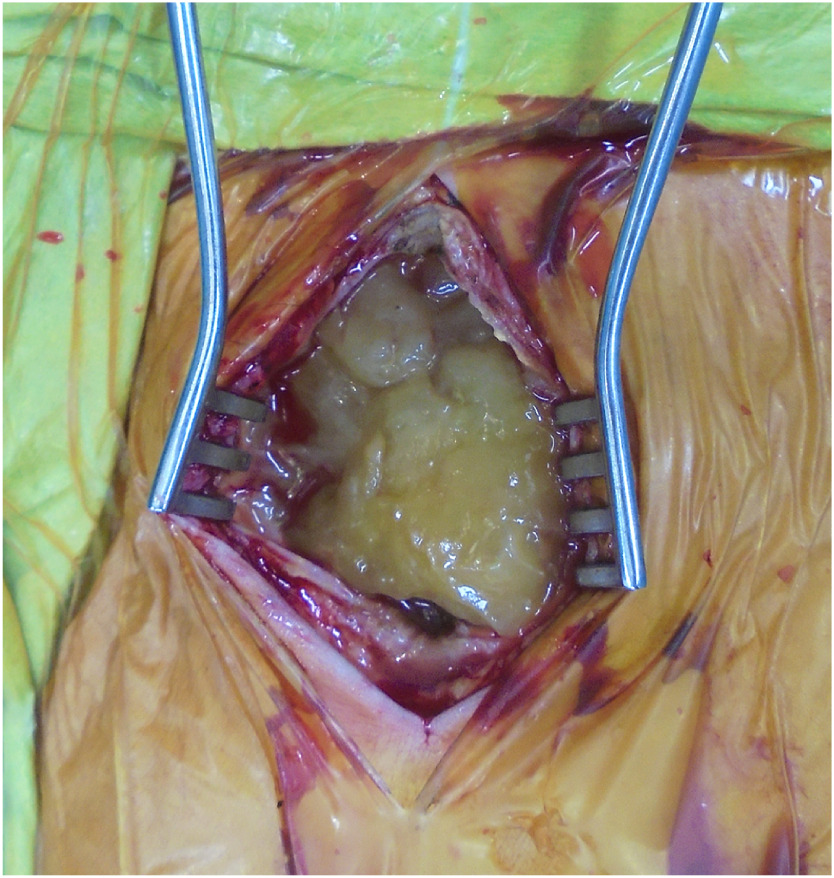
Fig. 2 Intraoperative findings of perigraft seroma. A gelatin-like large mass was detected after the longitudinal incision of the left groin. There were no signs of infection or bleeding. The upper side of the figure is the caudal side.

**Figure 3:**
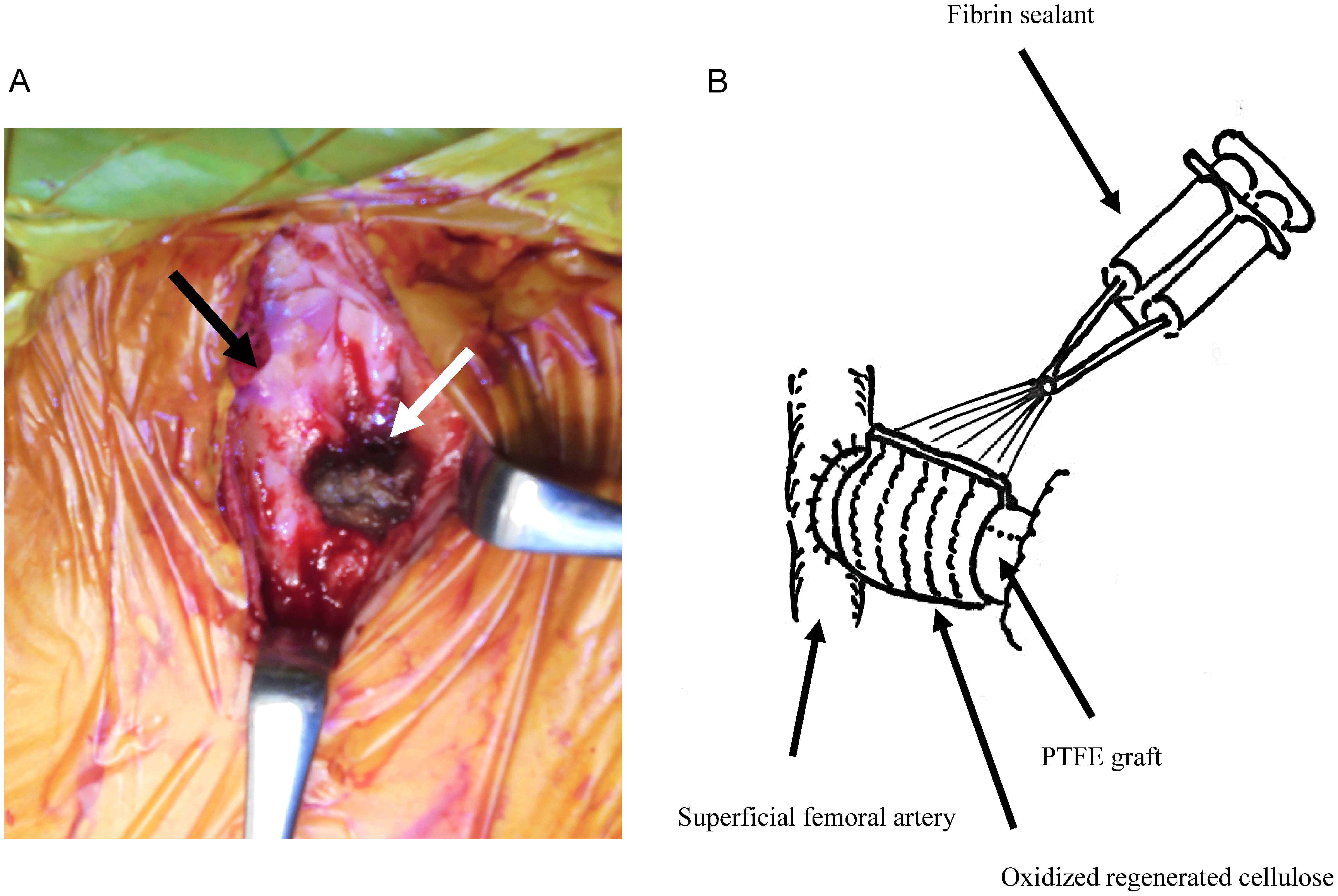
Fig. 3 Graft wrapping with two types of local hemostatic agents. (**A**) Intraoperative findings. Graft wrapping with oxidized regenerated cellulose and a sprayed fibrin sealant was conducted around the polytetrafluoroethylene (PTFE) graft just above the proximal site of anastomosis (white arrow). The black arrow shows the proximal side of the superficial femoral artery. The upper side of the figure is the caudal side. (**B**) Schema of graft wrapping. The PTFE graft just above the proximal site of anastomosis was tightly wrapped by oxidized regenerated cellulose, sprayed with fibrin sealant, and then fixed.

A 76-year-old female with end-stage renal disease receiving regular HD underwent the surgical creation of an arteriovenous fistula or AVG at the bilateral forearm or upper arm several times. A lower extremity HD access with left-thigh AVG; Advanta, 4- to 6-mm polytetrafluoroethylene (PTFE) tapered graft (Atrium Medical, Hudson, NH, USA) in the loop configuration was introduced approximately 18 months ago at our institute. The PTFE graft was a slider graft placed using a standard implantation technique that included multiple precautions to avoid excessive graft ultrafiltration, such as never forcing saline or blood into the graft under pressure and removing the distal venous-side clamp first.^6)^ Based on the product information, this PTFE graft has an average outer surface porosity of 50 µm, and an average inner surface porosity of 20 µm. Thigh loop AVG was perfused from the proximal side of the superficial femoral artery to the proximal side of the great saphenous vein (**Fig. 1**). The patency of thigh loop AVG was preserved without graft failure or other graft-related complications. However, progressively large and non-pulsatile local bulging at the left side of the groin developed 18 months after the creation of AVG. Although local compression was initially performed, bulging continued to increase. There were no signs of infection, such as fever, local skin rash, or a discharge from the groin. Lower extremity enhanced computed tomography showed a large low-density area measuring 47×42 mm around the AVG arterial side anastomosis to which the AVG graft was anastomosed with the superficial femoral artery, which was suspected to be PS. A pseudoaneurysm of the graft concomitantly developed at the tip of the AVG loop because the same area of AVG had been punctured several times. Therefore, surgery was considered. Laboratory data on admission revealed anemia with 9.8 g/dL of the hemoglobin concentration used to be seen in HD patients, and coagulation data was within the normal range, 243 mg/dL of fibrinogen concentration. Under general anesthesia, a longitudinal surgical incision was made at the left groin. The PTFE graft just below the proximal anastomosis of the superficial femoral artery was exposed, and the complete resection and removal of a large gelatin-like mass were performed (**Fig. 2**). There were no signs of infection, bleeding, or obvious serous leakage from the anastomosis. Difficulties were associated with intraoperatively detecting the location of serous leakage at the PTFE graft. After irrigation with warm saline solution, the exposed PTFE graft, with a length of approximately 2 cm, was wrapped using the local hemostatic agent, oxidized regenerated cellulose; Surgicel Nu-Knit (Johnson & Johnson Medical Ltd., Somerville, NJ, USA). Another local hemostatic agent, a fibrin sealant, Beriplast P (CSL Behring, Marburg, Germany) was then sprayed at this point to avoid further serous leakage from the PTFE graft. The exposed PTFE graft was completely wrapped using these local hemostatic agents and tightly packed (**Figs. 3A** and **3B**). Local PTFE graft interposition using the same 6-mm PTFE graft; Advanta (Atrium Medical, Hudson, NH, USA), with a short length of 25 mm, was concomitantly performed after the removal of the pseudoaneurysm. The incision at the left groin was tightly closed without a subcutaneous dead space to reduce the risk of PS recurrence. Surgery was completed without any serious complications, and the postoperative clinical course was fair. This vascular access has been used since the day after surgery, and there has been no local recurrence of PS for four months after surgery.

## Discussion

PS sometimes occurs after prosthetic graft implantation, and it may have a negative impact on the clinical outcome of a vascular graft, ultimately leading to graft failure. The development of PS after creating AVG as vascular access for HD is a rare complication with a prevalence of only 1.7%.^5)^ As far as we checked, there were few previous reports of the incidence of PS in patients with lower extremity AVG, which was 5% of lower extremity AVGs more evident than upper limb AVGs.^7)^ The reason for more incidents of PS with AVG placement at the lower extremity is suspected that subcutaneous dead space around prosthetic grafts might be more common in the groin area. To avoid AVG failure, surgical interventions are sometimes indicated for PS if it is refractory to treatment. Managing uncurable PS involves conservative therapy, such as local compression, followed by percutaneous drainage. However, this strategy may be ineffective and increase the risk of graft infection and failure. Surgical drainage with or without a graft’s primary or secondary replacement with a different material is also considered.^3)^

In this case, discrimination with lymphorrhea was needed. If this gelatinous component originated from lymphorrhea, bulging was located at both arterial and venous sides of anastomosis, and local bulging might be recognized just after surgical intervention. However, this bulging originated only from the arterial side of anastomosis and developed over 18 months after the creation of vascular access. Seroma almost invariably develops around the arterial portion of the graft in PTFE grafts placed for vascular access. Therefore, PS was suspected and diagnosed in this case.

Based on previous findings, we discuss optimal strategies for surgical management of PS. Zanow et al. reported successful treatment of PS by fibrin sealing of the outer surface of PTFE grafts to prevent leakage,^8)^ which was successful in 84% of patients. They suggested that the instillation of fibrin glue only into the space surrounding the affected graft was insufficient because it could not completely and stably coat the outer graft surface. In an experimental evaluation of fibrin glue for PTFE grafts, Tsuchida et al. concluded that its sealant effects on preventing late fluid leakage were inadequate.^9)^ In the present case, we wrapped the PTFE graft using two types of local hemostatic agents because only one hemostatic agent may be insufficient to completely seal the outer grafts, resulting in the recurrence of PS. We expected both local hemostatic agents’ synergistic effects to successfully seal the graft.

Based on previous studies, reviews, and summaries, we propose a strategy for PS at the vascular access for HD.^4)^ We recommend conservative therapy, such as local compression or percutaneous drainage, to reduce or prevent PS by inducing local adhesion, and reducing the subcutaneous dead space. The minimally invasive surgical approach of graft wrapping with local hemostatic agents, as described for the present case, may be performed if PS is not located at the puncture site of AVG. Graft replacement using a different graft material may be considered to prevent graft failure if PS is refractory to treatment. Although surgical interventions are a radical approach, our surgical technique may be an alternative option for treating refractory PS before the surgical replacement of a related prosthetic graft with a different material.

## Conclusion

We herein described a surgical case of PS that was successfully treated by local graft wrapping using two types of hemostatic agents.
